# Effectiveness of Probiotics, Prebiotics, and Synbiotics in Managing Insulin Resistance and Hormonal Imbalance in Women with Polycystic Ovary Syndrome (PCOS): A Systematic Review of Randomized Clinical Trials

**DOI:** 10.3390/nu16223916

**Published:** 2024-11-16

**Authors:** Darly Martinez Guevara, Sinthia Vidal Cañas, Isabela Palacios, Alejandra Gómez, María Estrada, Jonathan Gallego, Yamil Liscano

**Affiliations:** Grupo de Investigación en Salud Integral (GISI), Departamento Facultad de Salud, Universidad Santiago de Cali, Cali 5183000, Colombia; darly.martinez00@usc.edu.co (D.M.G.); sinthia.vidal00@usc.edu.co (S.V.C.); isabela.palacios00@usc.edu.co (I.P.); alejandra.gomez01@usc.edu.co (A.G.); maria.estrada03@usc.edu.co (M.E.); jhonatan.gallego00@usc.edu.co (J.G.)

**Keywords:** probiotics, insulin resistance, hormonal imbalance, polycystic ovary syndrome (PCOS), gut microbiota, synbiotics, metabolic health, women’s health, systematic review, randomized controlled trials

## Abstract

**Background/Objectives:** Polycystic ovary syndrome is a common endocrine disorder in women of reproductive age characterized by insulin resistance and hormonal imbalances. Recent research suggests that probiotics and synbiotics may improve these parameters by modulating the gut microbiota. This study systematically reviewed randomized clinical trials evaluating the impact of probiotic, prebiotic, and synbiotic supplementation on insulin resistance and hormonal parameters in women with PCOS. **Methods:** Exhaustive searches were conducted in PubMed, Cochrane CENTRAL, Scopus, Web of Science, and Embase, following PRISMA guidelines. Randomized trials assessing supplementation with probiotics, prebiotics, or synbiotics for at least 8 weeks in women diagnosed with PCOS according to the Rotterdam criteria were included. Data on participants, interventions, and outcomes related to insulin resistance and hormones were extracted. **Results:** Eleven studies from Iran involving overweight or obese women aged 15 to 48 were included. Probiotic and synbiotic supplementation showed significant improvements in insulin resistance (reductions in HOMA-IR, fasting glucose, and insulin), lipid profiles (decreased LDL and triglycerides; increased HDL), and hormonal balance (increased SHBG, decreased total testosterone). Synbiotics had more pronounced effects than probiotics or prebiotics alone. Adherence was high, and side effects were minimal. **Conclusions:** Despite promising results, limitations such as small sample sizes, homogeneous populations, and short intervention durations limit the generalization of the findings. Larger, longer, multicenter trials with diverse populations and standardized methodologies are needed to confirm the efficacy and safety of synbiotics in managing PCOS. Integrating these interventions could improve clinical management and quality of life for affected women, but additional evidence is required to support widespread use.

## 1. Introduction

Polycystic ovary syndrome (PCOS) is a prevalent endocrine disorder affecting 5% to 10% of women of reproductive age, characterized by hyperandrogenism, ovulatory dysfunction, and a polycystic ovarian morphology [1,2]. Diagnosis is commonly based on the Rotterdam criteria, which require at least two of the following: oligo- or anovulation, clinical and/or biochemical signs of hyperandrogenism, and a polycystic ovarian morphology detected by ultrasound [3,4,5]. PCOS phenotypes (A–D) vary in metabolic profiles and risks. Hyperandrogenic phenotypes (OD-HA and HA-PCOM) exhibit adverse metabolic profiles, including elevated body mass index (BMI), higher waist-to-hip ratio, insulin resistance, and dyslipidemia, increasing the risk of metabolic syndrome, type 2 diabetes mellitus, and cardiovascular diseases [6,7,8]. Phenotypes lacking hyperandrogenism generally present more favorable metabolic profiles, though some normoandrogenic women may still face metabolic challenges.

PCOS is associated with various metabolic complications, including a higher prevalence of metabolic syndrome, reaching up to 56% in some studies [9,10,11]. Insulin resistance is particularly common, affecting approximately 60% to 75% of women with PCOS when assessed using the Homeostasis Model Assessment for Insulin Resistance (HOMA-IR) [12,13,14]. Hyperinsulinemia exacerbates hyperandrogenism by decreasing sex hormone-binding globulin (SHBG), increasing free testosterone levels, and worsening clinical manifestations such as hirsutism, acne, irregular menstrual cycles, and infertility [15,16]. Insulin directly stimulates ovarian theca cells to increase testosterone production and affects key enzymes in androgen synthesis [16,17,18,19].

Current treatments involve combined hormonal contraceptives to regulate menstrual cycles and reduce androgen effects [20,21]. However, these may worsen insulin resistance, alter glucose metabolism, and negatively affect cardiovascular profiles by increasing triglyceride levels and systemic inflammation markers [22,23]. Thus, while they alleviate some PCOS symptoms, they may contribute to metabolic complications, highlighting the need for more personalized treatments [24,25].

Recent research suggests the gut microbiota significantly influences metabolic and hormonal pathways associated with PCOS. Dysbiosis may contribute to chronic low-grade inflammation, exacerbating insulin resistance and hormonal imbalances [26]. Women with PCOS show decreased diversity and altered abundance of specific bacterial taxa compared to healthy controls [27,28,29].

Similarly, alterations in gut microbiota have been linked to type 2 diabetes, with certain bacterial genera positively or negatively correlated with the disease [30,31]. Probiotics, particularly *Lactobacillus* and *Bifidobacterium* species, have emerged as potential biotherapeutics for managing insulin resistance and metabolic disorder [32,33].

Consequently, prebiotics, probiotics, and synbiotics have gained attention as potential treatments for PCOS by modulating gut microbiota. Probiotics are live microorganisms that confer health benefits when consumed in adequate amounts, while prebiotics are non-digestible substances that promote the growth of beneficial microbes [34,35]. Synbiotics combine both to enhance the survival and implantation of beneficial microorganisms. These interventions may address microbiota imbalances, inflammation, insulin resistance, lipid profiles, and hormonal dysregulation more effectively than traditional treatments [36].

Multiple randomized controlled trials (RCTs) have assessed the role of probiotics and synbiotics in women with PCOS, reporting improvements in insulin resistance markers, reductions in androgen levels, and favorable shifts in lipid profiles [37,38,39,40]. However, study designs, bacterial strains, dosages, and intervention durations vary significantly, making it difficult to compare results and establish guidelines. Many studies lack rigorous methodological design and are often small-scale. Additionally, diagnostic criteria like the Rotterdam criteria include patients with diverse phenotypes, contributing to heterogeneity.

Uncertainties remain about how probiotics and synbiotics exert beneficial effects in PCOS patients. Proposed mechanisms include regulation of gut barrier integrity, suppression of systemic inflammation, improvement of insulin signaling pathways, and effects on the hypothalamic–pituitary–ovarian axis [41,42]. More research is needed to establish precise mechanisms, optimal bacterial strains, dosages, and treatment durations.

Considering these factors, the present systematic review aims to critically evaluate and synthesize existing RCTs focused on the effects of probiotics and synbiotics on insulin resistance and hormonal markers in women with PCOS. We hypothesize that probiotic and synbiotic interventions significantly improve insulin sensitivity and reduce androgen levels compared to control groups. This review seeks to provide insights into improving the pathophysiological understanding and personalized treatment management of PCOS.

## 2. Materials and Methods

This systematic review was conducted in accordance with the Preferred Reporting Items for Systematic Reviews and Meta-Analyses (PRISMA) guidelines [43]. The methodology was designed to ensure a comprehensive and unbiased selection of relevant studies evaluating the effectiveness of probiotics and synbiotics in managing insulin resistance and hormonal imbalance in women with PCOS.

In women with PCOS (P), how does probiotic or prebiotic supplementation, or the supplementation of both (I), compared to placebo or standard treatments (C), affect insulin resistance and hormonal balance, thereby influencing metabolic health and quality of life (O)?

This study’s Prospero registration number is CRD42024587531.

### 2.1. Eligibility Criteria

The eligibility criteria for this review are detailed in Table 1 below.

### 2.2. Information Sources and Search Strategy

A comprehensive search of electronic databases was conducted to identify relevant studies. The databases searched included the following:

- PubMed;
- Cochrane Central Register of Controlled Trials (CENTRAL);
- Scopus;
- Web of Science;
- Embase.

The data were organized using Zotero version 6.0 (accessed on 24 August 2024).

### 2.3. Search Algorithm

The search strategy combined Medical Subject Headings (MeSH) and free-text terms related to PCOS, probiotics, prebiotics, synbiotics, insulin resistance, and hormonal parameters. Boolean operators (AND, OR) were used to refine the search. An example of the search strategy used in PubMed is as follows:

(“polycystic ovary syndrome” OR “PCOS”) AND (probiotic OR prebiotic OR synbiotic) AND (insulin OR “insulin resistance” OR “HOMA-IR” OR “pancreatic β cell function” OR “C reactive protein” OR “hormonal status” OR “testosterone” OR “androgens”)

This search algorithm was adapted for each database according to its specific search functionalities. Additional searches were conducted by reviewing the reference lists of relevant articles to identify any studies that might have been missed.

### 2.4. Study Selection

Two reviewers (D.M.G. and S.V.C.) independently screened the titles and abstracts of all identified articles for eligibility. Full-text articles were retrieved for studies that appeared to meet the inclusion criteria or if eligibility was unclear from the abstract. Discrepancies between the reviewers were resolved through discussion, and if necessary, a third reviewer (Y.L.) was consulted to reach a consensus.

### 2.5. Data Extraction

Two reviewers (D.M.G. and S.V.C.) independently extracted information from the primary studies using a standardized data extraction form. The extracted data included details of the clinical trial:

- Study characteristics: first author, publication year, country, and study design.
- Participant characteristics: number of participants, age, BMI, sex (all female participants), and diagnostic criteria for PCOS.
- Intervention details: type of probiotic/prebiotic/synbiotic used, bacterial strains, dosage, form of administration, and duration of intervention.
- Comparator: details of the control group (placebo or standard care).
- Outcomes measured: primary outcomes (e.g., insulin resistance markers, hormonal parameters), secondary outcomes (e.g., lipid profile, inflammatory markers), and methods of measurement.
- Results: main findings, statistical significance, and conclusions drawn by the authors.
- Limitations: as reported by the authors.

Subsequently, a third reviewer (Y.L.) verified the integrity and accuracy of the recorded information.

**Figure 1:**
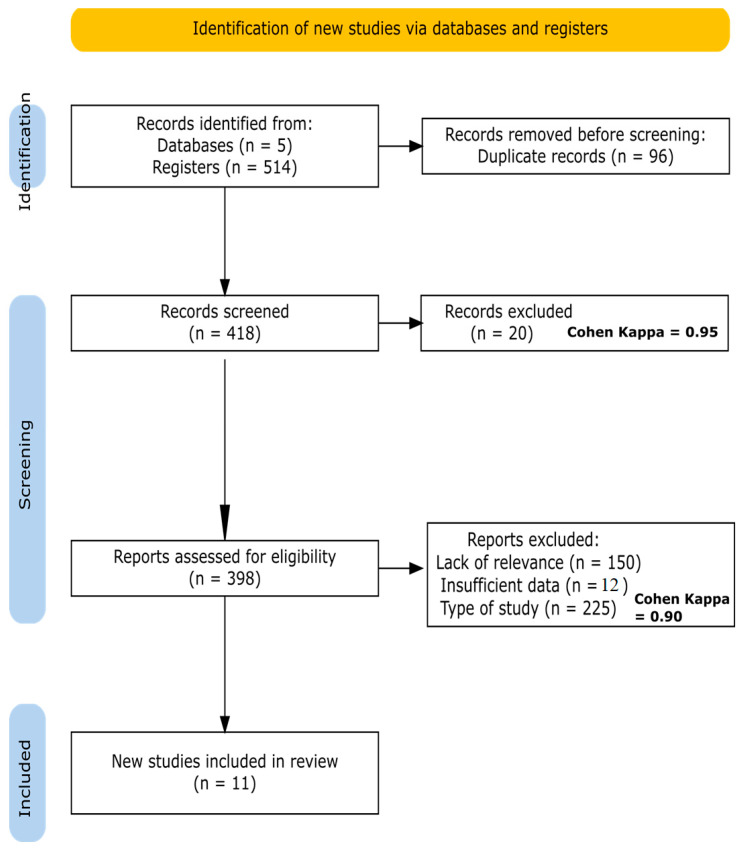
PRISMA flowchart. A Cohen’s kappa of 0.95 and 0.82 indicates a high level of agreement between reviewers, ensuring the validity of the results.

**Figure 2:**
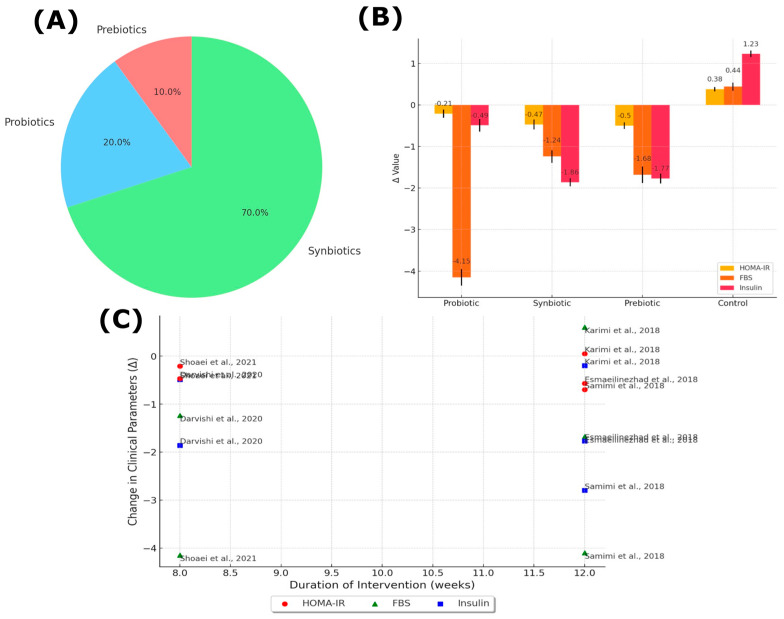
Clinical outcomes of prebiotic, probiotic, and synbiotic interventions in patients with PCOS. (**A**) Distribution of prebiotics, probiotics, and synbiotics used in interventions. (**B**) Changes in HOMA-IR, FBS, and insulin by intervention group. HOMA-IR (Homeostatic Model Assessment for Insulin Resistance) is marked in red. FBS (fasting blood sugar) is in orange. Insulin is in yellow. The bars reflect how each parameter changed based on the type of supplement used (probiotic, synbiotic, prebiotic, and control). (**C**) Relationship between duration of intervention and change in clinical parameters (Δ). Red dots represent changes in HOMA-IR. Green dots represent changes in FBS. Blue dots represent changes in insulin [38,39,40,45,46].

Figure 1 was created with the online R package PRISMA2020 [44] (https://estech.shinyapps.io/prisma_flowdiagram/, accessed on 24 August 2024). Figure 2 was created using R software package ggplot2, version 4.3.0 (https://cran.r-project.org/bin/windows/base/old/4.3.0/, accessed on 24 August 2024).

### 2.6. Risk-of-Bias Assessment

The risk-of-bias assessment for the included studies was conducted independently by two reviewers (D.M.G. and S.V.C.) using the Cochrane Risk of Bias Tool for Randomized Trials [47], with data entered into Review Manager version 5.4^®^ (RevMan, The Cochrane Collaboration, accessed on 24 August 2024). The following domains were evaluated:

- Random sequence generation (selection bias);
- Allocation concealment (selection bias);
- Blinding of participants and personnel (performance bias);
- Blinding of outcome assessment (detection bias);
- Incomplete outcome data (attrition bias);
- Selective reporting (reporting bias).

For each domain, studies were assessed as having a low, high, or unclear risk of bias based on predetermined guidelines. Discrepancies in the risk-of-bias assessment were resolved through discussion between the reviewers, and if necessary, a third reviewer (Y.L.) was consulted.

To confirm the consistency of the evaluation process, a subset of the studies was reassessed, and Cohen’s kappa coefficient was computed using IBM SPSS Statistics version 27.0 (IBM Corp., Armonk, NY, USA; accessed on 24 August 2024) to quantify the agreement between reviewers.

### 2.7. Data Synthesis

Due to the heterogeneity among the included studies in terms of interventions, bacterial strains, dosages, and outcomes measured, a qualitative synthesis was conducted. The results are presented in narrative form, accompanied by tables summarizing the key characteristics and findings of the studies.

### 2.8. Ethical Considerations

As this study is a systematic review of the published literature, it did not involve direct interaction with human subjects or animals and thus did not require ethical approval.

## 3. Results

### 3.1. Characteristics of the Included Studies

A total of 514 records were identified across five databases, with 96 duplicates removed, leaving 418 records. Of these, 20 were excluded based on title and abstract screening, using a peer review process with a Cohen’s kappa of 0.95, indicating excellent agreement between the reviewers. Subsequently, 398 records were assessed for eligibility through full-text reviews. Among these, 150 records were excluded for not meeting relevance criteria, 12 due to insufficient data, and 225 because they did not match the required study type. The Cohen’s kappa for this phase was 0.90, also reflecting excellent agreement. As a result, 11 articles, all conducted in Iran, were ultimately included in this systematic review, as shown in Figure 1.

### 3.2. Overview of Study Results

Table 2 below outlines the general characteristics of the 11 studies included in this review. All studies were randomized clinical trials, with seven being double-blind and four triple-blind, and all were conducted in Iran among women of reproductive age. The consistent application of the Rotterdam criteria ensured the uniform selection of patients, thereby facilitating more robust comparisons across the different studies. This uniform selection was crucial for minimizing variability in patient populations, ensuring that all participants met standardized diagnostic criteria for PCOS. Consequently, the studies could be more reliably compared, allowing for a clearer understanding of the interventions’ effects on metabolic and hormonal outcomes in PCOS.

### 3.3. Participant Characteristics

Table 3 details the characteristics of the participants in the reviewed studies. All studies exclusively included women, with sample sizes ranging from 23 to 45 participants. The ages of the participants varied between 15 and 48 years, and the BMI generally ranged from 25 to 40 kg/m^2^, classifying most participants as overweight or obese.

For example, Esmaeilinezhad et al., 2018 [38] included 92 women, evenly divided into four groups of 23 participants each: synbiotic pomegranate juice, pomegranate juice, synbiotic beverage, and control. The baseline parameters measured included HOMA-IR, fasting blood glucose (FBS), insulin levels, and Quantitative Insulin Sensitivity Check Index (QUICKI). In another study, by Shoaei et al., 2021 [39], 72 women participated, with 36 in the probiotic group and 36 in the placebo group. The ages ranged from 15 to 40 years, and the BMI was between 25 and 30 kg/m^2^. The study evaluated HOMA-IR, FBS, and insulin as baseline parameters.

Similarly, Darvishi et al., 2020 [40] included 68 women, divided into two groups of 34 each (synbiotic and placebo), with ages between 20 and 44 years and a BMI of 25 kg/m^2^ or higher. The recorded baseline parameters were HOMA-IR, FBS, insulin, and HDL cholesterol. Other studies, such as Karimi et al., 2018 [50] and 2020 [51], Samimi et al., 2018 [46], and Arab et al., 2022 [49], followed similar structures, focusing on different metabolic and hormonal markers according to each study’s specific objectives.

In particular, Karimi et al., 2018 [50] and 2020 [51] included 30 and 44 women, respectively, evaluating parameters such as SHBG, total testosterone, mF-G scores, hs-CRP, TAC, and malondialdehyde (MDA). Samimi et al., 2018 [46] and Gholizadeh Shamasbi et al., 2018 [48] also incorporated measurements of fasting glucose, insulin, HOMA-IR, triglycerides, VLDL, AIP, and various inflammatory and hormonal markers. Lastly, Arab et al., 2022 [49] assessed 45 women in the intervention group and 43 in the control group, measuring SHBG, total testosterone, FAI, and DHEA-S.

### 3.4. Intervention Details and Comparison Groups

The interventions across the reviewed studies encompassed a variety of probiotic, prebiotic, and synbiotic formulations designed to address metabolic and hormonal disturbances in women with PCOS. These interventions varied in strains, dosages, forms, and colony-forming units (CFUs), all of which could influence their effectiveness (See Table 4).

For instance, studies like Esmaeilinezhad et al., 2018 [38] and Esmaeilinezhad et al., 2019 [37] utilized synbiotic pomegranate juice, with participants consuming either 2 L per week or 300 mL daily over periods ranging from 8 to 12 weeks. These interventions were compared to placebo pomegranate juice or flavored water, allowing for a direct assessment of the combined effects of probiotics and prebiotics in a liquid medium. The juice form likely enhances metabolic effects by providing both probiotic benefits and the antioxidant properties of pomegranate.

Conversely, several studies employed capsule-based interventions. Shoaei et al., 2021 [39] and Karimi et al., 2018 [45] administered multi-strain probiotics in capsule form, typically containing strains such as *Lactobacillus casei*, *L. rhamnosus*, and *Bifidobacterium longum*, delivered in daily doses ranging from 500 mg to 1000 mg over 8 to 12 weeks. Additionally, studies like Darvishi et al., 2020 [40] and Gholizadeh Shamasbi et al., 2018 [48] combined probiotics with prebiotics such as inulin or dextrin in capsule form to enhance gut flora and overall metabolic health. The prebiotics served as fuel for the probiotics, potentially increasing their efficacy in modulating the gut microbiota and improving insulin sensitivity.

Synbiotic capsules were also utilized in studies by Karimi et al., 2020 [51] and Nasri et al., 2018 [52], where multi-strain synbiotics were paired with prebiotics like inulin, administered as two 500 mg doses daily over 12 weeks. These interventions ensured a controlled dosage, providing participants with a precise amount of beneficial bacteria and prebiotics. The control groups in these studies typically received placebo capsules containing starch or maltodextrin, allowing for clear comparisons between active and inactive treatments.

CFUs varied significantly across studies, influencing the potential efficacy of the interventions. Studies such as Arab et al., 2022 [49] and Karamali et al., 2018 [50] employed high CFU counts, ranging from 10^8^ to 10^10^ CFU/g for each probiotic strain, potentially enhancing the probiotic effects compared to studies with lower CFU counts like Nasri et al., 2018 [52]. Dosages also varied, from 500 mg to 1000 mg per capsule, with some studies administering multiple capsules daily to achieve the desired intake of probiotics and prebiotics.

The duration of interventions was consistent across most studies, typically lasting between 8 and 12 weeks, which is generally sufficient to observe meaningful changes in metabolic and hormonal parameters. All studies included placebo comparison groups to ensure that observed effects could be attributed to the active interventions. Placebos varied in form, including starch, maltodextrin, flavored water, or maltodextrin-diluted powders, depending on the study design.

Despite the variations in probiotic and synbiotic strains, dosages, forms, and CFU counts, all studies aimed to improve metabolic and hormonal outcomes in women with PCOS. The diversity in intervention formulations underscores the complexity of determining the most effective probiotic or synbiotic regimen, as different delivery methods and strain combinations could lead to varying clinical outcomes. Future research should aim to standardize these variables to better compare and understand the efficacy of different formulations.

The reviewed studies present mixed but promising results regarding the effects of probiotics, prebiotics, and synbiotics on insulin resistance, hormonal markers, and metabolic profiles in women with PCOS. Commonly analyzed metrics included HOMA-IR, fasting blood glucose, and hormone levels such as testosterone and SHBG.

Esmaeilinezhad et al., 2018 [38] (see Table 5) demonstrated that synbiotic pomegranate juice significantly reduced HOMA-IR, fasting insulin, and glucose, indicating moderate improvements in insulin sensitivity compared to pomegranate juice or synbiotics alone. Despite high adherence rates, the study was limited by its small sample size and lack of long-term follow-up. Similarly, Esmaeilinezhad et al., 2019 [37] reported significant improvements in lipid profiles and cardiovascular markers with synbiotic pomegranate juice, though they did not measure body composition or gut microbiota changes.

Shoaei et al., 2021 [39] found no significant reduction in HOMA-IR; however, a deeper analysis revealed a potential reduction in insulin levels, which may have been obscured by the study’s short duration. Darvishi et al., 2020 [40] noted improvements in glycemic indices and HDL cholesterol with synbiotics, though the absence of gut microbiota data limited mechanistic insights. Conversely, Karimi et al., 2018 [45] observed no significant changes in HOMA-IR or FBS but found a decrease in apelin 36, suggesting an anti-inflammatory effect rather than direct metabolic impacts.

Samimi et al., 2018 [46] highlighted synbiotics’ positive effects on insulin sensitivity and lipid metabolism, although total cholesterol and LDL levels remained unchanged. Gholizadeh Shamasbi et al., 2018 [48] demonstrated prebiotics’ ability to lower LDL, total cholesterol, and inflammatory markers, while also reducing free testosterone, indicating potential benefits for hyperandrogenism. Arab et al., 2022 [49] and Karamali et al., 2018 [50] focused on hormonal regulation, showing mixed results in clinical symptoms of hyperandrogenism but clear improvements in SHBG and testosterone levels.

Karimi et al., 2020 [51] and Nasri et al., 2018 [52] supported the positive effects of synbiotics on lipid profiles and inflammation, although further research with longer follow-ups is needed to confirm these findings across diverse populations. Across the studies, adherence rates were generally high, and side effects were minimal, with only a few instances of mild allergies reported.

Key findings across the studies include significant reductions in HOMA-IR, improvements in lipid profiles, enhanced hormonal balance, and reductions in inflammatory markers. However, limitations such as small sample sizes, short durations, and the absence of comprehensive gut microbiota assessments were common. For instance, Esmaeilinezhad et al., 2018 [38] and Darvishi et al., 2020 [40] were limited by their small sample sizes and lack of long-term follow-up, while studies like Arab et al., 2022 [49] and Nasri et al., 2018 [52] faced challenges such as short durations and non-standardized measurement methods.

Figure 2 is organized into three sections: the distribution of intervention types, their impact on specific clinical markers, and the relationship between intervention duration and achieved effects.

In Figure 2A, a pie chart illustrates the distribution of intervention types across the studies. Notably, combined probiotics and prebiotics (synbiotics) were the most frequently used interventions, comprising 70% of the total. Probiotics alone accounted for 20%, while prebiotics alone represented only 10%. This distribution highlights a strong preference for synbiotic treatments, likely due to the anticipated synergistic benefits of combining probiotics with prebiotics for broader metabolic improvements.

Figure 2B depicts the changes in three key clinical parameters related to insulin resistance: HOMA-IR, FBS, and insulin levels. Synbiotics demonstrated the most significant impact, with notable reductions in all three parameters. Specifically, HOMA-IR decreased by −0.57, indicating enhanced insulin sensitivity. Additionally, the synbiotic group showed the greatest reductions in both insulin and FBS, underscoring its potential effectiveness. Probiotic interventions also yielded improvements, albeit to a lesser extent, with moderate reductions in HOMA-IR and insulin levels. Prebiotics alone had a smaller yet beneficial effect. In contrast, the placebo group experienced an increase in HOMA-IR by +1.23, reflecting a rise in insulin resistance without any therapeutic intervention.

Lastly, Figure 2C explores the relationship between the duration of the interventions and the changes in clinical outcomes. The data reveal that longer intervention periods, typically around 12 weeks, are associated with greater improvements in HOMA-IR, FBS, and insulin levels. This suggests that extended treatment durations are crucial for achieving substantial metabolic benefits. While shorter interventions, usually lasting 8 weeks, also resulted in improvements, the effects were less pronounced compared to longer durations. Therefore, the figure underscores the importance of sustained treatment to maximize improvements in insulin sensitivity and glycemic control.

Overall, Figure 2 emphasizes the superior effectiveness of synbiotic interventions in improving metabolic markers in women with PCOS, particularly when administered over longer periods. The preference for combined probiotic and prebiotic treatments, along with the observed dose–response relationship between intervention duration and metabolic outcomes, highlights the potential of synbiotics as a comprehensive approach to managing PCOS-related metabolic disturbances.

### 3.5. Risk-of-Bias Assessment Results

**Figure 3:**
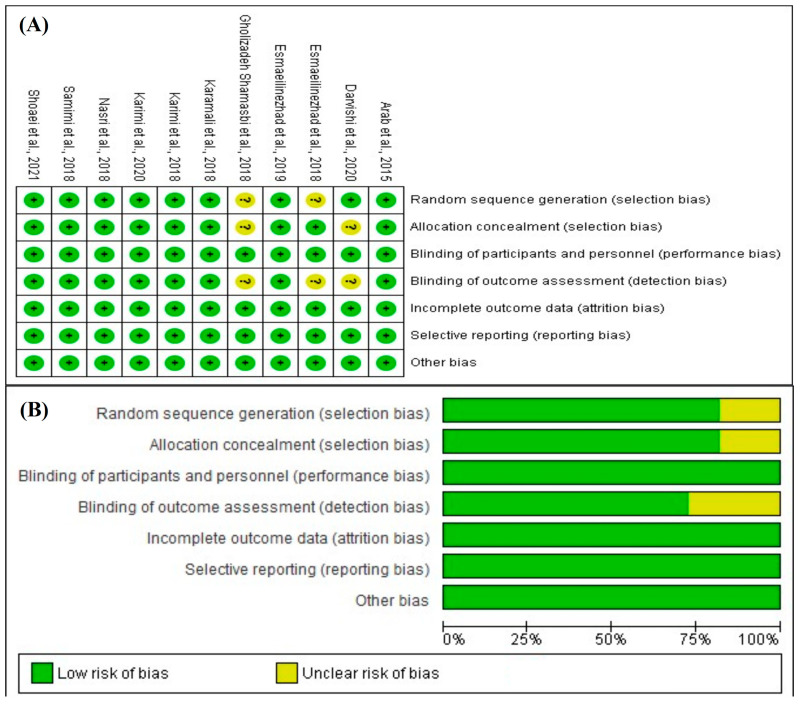
Cochrane risk-of-bias assessment for randomized studies of interventions in this systematic review. (**A**) Risk-of-bias summary: Review of the authors’ judgments about each risk-of-bias item for each included study. The symbol “+” indicates a low risk of bias, the symbol “?” indicates an unclear risk of bias. The colors used are green for low risk of bias, yellow for unclear risk of bias [37,38,39,40,45,46,48,49,50,51,52]. (**B**) Risk-of-bias graph: Review of the authors’ judgments about each risk-of-bias item presented as percentages across all included studies. Figure created by RevMan 5 (accessed on 24 August 2024).

The risk-of-bias assessment for the studies, taken from the RevMan 5.4 analysis, shows that the overall quality of the included trials was robust. Most domains in Figure 3 present a low risk of bias, and this is especially true for domains like the generation of random sequences, where almost all studies had appropriate randomization methods to avoid selection bias. Allocation concealment was also ingeniously handled, as most of the studies scored as having low risk, meaning the actual process of allocating participants to either intervention or control was well-blinded to reduce selection bias. However, in some instances, allocation concealment was rated as unclear due to the need for further explicit reporting in the studied papers about how this process was conducted; hence, a slight doubt on selection bias remained.

Performance bias, or blinding of participants and personnel, was a less easily surmountable problem. A number of this review’s component studies, including many of the most robustly designed, were considered to be at high risk. This was largely due to the inherent nature of the clinical trials in this field, since maintaining blinding for participants and personnel was difficult because of obvious differences between active interventions (probiotics, synbiotics, or prebiotics) and placebos. Lack of blinding may introduce performance bias, which could potentially influence the behavior of participants or the expectations of the researchers [53].

On the other hand, blinding of outcome assessment or detection bias was generally well managed in most studies. In most trials, the assessors of clinical outcomes were blinded to the intervention groups, which minimized the occurrence of biased measurements of outcome. Attrition bias or incomplete outcome data showed consistent low risk across the studies. Most trials reported participant dropouts and addressed missing data adequately, with no studies displaying a high risk of bias in this domain. Selective reporting was consistently rated as low risk across all studies, indicating comprehensive reporting of both positive and negative outcomes. The studies demonstrated minimal external factors influencing the results, as reflected by the low risk of other biases.

When we combine these results with the risk-of-bias assessment using the Jadad scale (see Table 6), it is clear that the included studies are methodologically strong and have minimal risk of bias that could undermine their findings. Every study scored a perfect 5 out of 5 on the Jadad scale, demonstrating excellent quality. This top score highlights their rigorous randomization, effective blinding, and transparent handling of withdrawals and dropouts, all of which make their results more reliable and trustworthy.

## 4. Discussion

### 4.1. Main Findings

This systematic review sought to answer the following question: How does supplementation with probiotics, prebiotics, or synbiotics, compared to placebo or standard treatments, affect insulin resistance and hormonal balance in women with PCOS, influencing their metabolic health and quality of life? The relevance of this study lies in the need to identify effective and safe interventions that address the characteristic metabolic and hormonal imbalances of PCOS, a condition that significantly impacts the quality of life of women of reproductive age.

The results demonstrated multiple benefits associated with probiotic and synbiotic-based interventions in women with PCOS. Significant reductions were observed in the HOMA-IR index, indicating substantial improvements in insulin sensitivity. Studies such as those by Esmaeilinezhad et al., 2018 [38], Shoaei et al., 2021 [39], and Samimi et al., 2018 [46] reported notable decreases in fasting glucose and insulin, reflecting better metabolic function.

Additionally, there were improvements in lipid profiles, with reductions in LDL cholesterol and triglycerides and increases in HDL cholesterol. Research by Esmaeilinezhad et al., 2019 [37] and Karimi et al., 2020 [51] supports these findings, suggesting a decrease in the cardiovascular risk associated with PCOS.

Regarding hormonal balance, increases in SHBG levels and decreases in total testosterone were recorded, suggesting a reduction in the hyperandrogenemia typical of PCOS. Studies such as those by Arab et al., 2022 [49] and Karamali et al., 2018 [50] demonstrated improvements in clinical symptoms of hyperandrogenism, which could translate into a better quality of life for patients. However, the limited number of studies assessing SHBG and hirsutism requires careful interpretation of these findings, as the evidence is not yet conclusive. Future research should aim to clarify the effect of probiotics and synbiotics on SHBG and clinical symptoms such as hirsutism to better understand the therapeutic potential of these interventions in managing hyperandrogenism in PCOS. According to the meta-analysis by Shamasbi et al., 2020 [54], the use of probiotics and synbiotics in women with PCOS led to a significant increase in SHBG levels compared to the placebo group, suggesting an improvement in the hormonal profile. However, regarding hirsutism symptoms, the same study did not find significant differences between the intervention and control groups. This indicates that probiotics and synbiotics may not have a direct impact on reducing hirsutism, and their effect on this condition remains inconclusive based on current evidence.

Reductions were also observed in inflammatory markers like hs-CRP, highlighting the anti-inflammatory potential of these interventions [45,52]. Reducing systemic inflammation is crucial given its role in the pathophysiology of PCOS and its impact on overall metabolic health.

It is important to note that interventions with synbiotics consistently showed the most significant benefits compared to probiotics and prebiotics administered separately. Combinations of probiotics and prebiotics offered more robust and comprehensive improvements, possibly due to the synergy between both components that enhances modulation of the gut microbiota and optimizes metabolic and hormonal functions [38,40].

The most commonly used probiotic strains included *Lactobacillus acidophilus*, *Lactobacillus casei*, *Lactobacillus rhamnosus*, *Bifidobacterium longum*, *Streptococcus thermophilus*, and *Bacillus coagulans.* The most common pharmaceutical forms were capsules and synbiotic juices, with durations ranging between 8 and 12 weeks. Synbiotic combinations produced more pronounced effects on both insulin resistance and hormonal balance.

Finally, the high adherence rate and minimal reported side effects reinforce the viability and safety of these interventions. Most studies reported adherence rates above 90%, and adverse effects were insignificant or nonexistent [38,39]. This suggests that probiotics and synbiotics can be effectively integrated into existing treatment regimens, offering a viable alternative to conventional pharmacological therapies with lower associated risks.

### 4.2. Comparison with Previous Studies

Our findings are consistent with previous research highlighting the beneficial effects of probiotics, prebiotics, and synbiotics on metabolic and hormonal parameters in women with PCOS. Meta-analyses and systematic reviews have demonstrated improvements in insulin resistance, lipid profiles, and hormonal balance.

For example, Miao et al., 2021 [55] concluded that supplementation with probiotics and synbiotics improved insulin resistance in women with PCOS, supporting the idea that modulation of the gut microbiota positively influences metabolic and endocrine functions. Musazadeh et al., 2023 [56] found that synbiotics significantly improved lipid profiles and anthropometric parameters, suggesting a beneficial effect on the management of obesity and related disorders.

Studies such as those by Cozzolino and Vitagliano, 2019 [57] and Karamali et al., 2018 [50] reported that probiotic supplementation was associated with improvements in metabolic, hormonal, and inflammatory parameters. Probiotics have been found to increase levels of SHBG, decrease total testosterone, and reduce hs-CRP and MDA. Synbiotic supplementation also showed beneficial effects on SHBG and inflammatory markers (Nasri et al., 2018 [52]).

Although some research, such as that by Angoorani et al., 2023 [41], suggests that probiotics are more effective than synbiotics on certain parameters, our findings indicate that synbiotics may have a broader impact on metabolism. These discrepancies highlight the need for more research to determine the most effective intervention and under what circumstances.

Inconsistencies in the literature underscore the importance of strain specificity, dosage, and treatment duration. McFarland et al., 2018 [58] emphasized that different strains within the same species can have variable effects on health, underscoring the need for specific clinical guidelines. Our study addresses this gap by identifying probiotic strains that show greater efficacy in improving metabolic and hormonal parameters in women with PCOS.

### 4.3. Impact of Probiotics on Insulin Resistance and Hormonal Balance in Polycystic Ovary Syndrome

Insulin resistance is fundamental in the pathogenesis of PCOS [59]. There is a link between gut microbiota dysbiosis and the development of PCOS, especially related to insulin resistance and obesity [60]. In this context, probiotics and synbiotics have shown promising effects in improving insulin sensitivity.

These microorganisms modify the composition and diversity of the gut microbiota, restoring a healthy balance of bacteria that positively impacts metabolic and hormonal pathways. Strains such as *Lactobacillus* and *Bifidobacterium* have demonstrated improvements in gut dysbiosis, correlating with more favorable sexual hormone levels and metabolic parameters [59].

Probiotics increase the production of short-chain fatty acids (SCFAs) like butyrate, propionate, and acetate, which have anti-inflammatory properties and improve insulin sensitivity. SCFAs maintain the integrity of the intestinal barrier and modulate immune responses [59,61,62], activate G protein-coupled receptors, and promote the release of peptides such as GLP-1 and PYY, contributing to glucose homeostasis [63].

Reducing systemic inflammation is another key mechanism. Chronic low-grade inflammation in PCOS interferes with insulin signaling. Probiotics decrease pro-inflammatory cytokines like TNF-α and IL-6, strengthen the intestinal barrier, and reduce the translocation of lipopolysaccharides into the bloodstream, improving insulin sensitivity [54].

Additionally, probiotics can influence bile acid metabolism, which acts as signaling molecules in glucose and lipid metabolism through receptors like FXR and TGR5 [64]. By modifying this metabolism, they enhance metabolic health [65]. In vitro studies have shown that probiotic complexes can alter bile acid profiles and microbial composition, increasing beneficial bacteria and reducing harmful metabolites [66].

Regarding hormonal regulation, probiotics can affect levels of sex hormones such as testosterone, LH, and FSH. Strains of *Lactobacillus* and *Bifidobacterium* have demonstrated reductions in testosterone and improvements in hormonal profiles, possibly mediated by the gut–brain axis and microbial modulation of hormonal metabolism [54,59]. By improving insulin resistance, they reduce hyperinsulinemia, decreasing ovarian androgen production and increasing levels of SHBG, which reduces free testosterone [67].

Probiotics also enhance antioxidant capacity by elevating total glutathione and total antioxidant capacity, mitigating oxidative stress in PCOS [54]. They improve lipid profiles by reducing triglycerides, total cholesterol, and LDL, benefiting the management of associated dyslipidemia [68,69].

Clinical evidence supports these mechanisms. Studies have reported significant reductions in fasting glucose, insulin levels, and HOMA-IR in women with PCOS receiving probiotic and synbiotic supplementation [55]. Decreases in testosterone, increases in SHBG, and improvements in symptoms like hirsutism and menstrual irregularities were also observed [50].

Therefore, probiotics positively impact insulin resistance and hormonal balance in PCOS by modulating the gut microbiota, increasing SCFAs, reducing systemic inflammation, and improving insulin signaling. These combined effects contribute to better glycemic and hormonal control, playing a crucial role in the comprehensive management of PCOS.

### 4.4. Limitations of the Included Studies

Despite these promising findings, it is essential to consider the limitations and risk of bias present in the reviewed studies to interpret the results accurately.

A key limitation is the small sample size in most studies, which can decrease statistical power and increase the risk of type II errors [29,70]. Additionally, all studies were conducted in Iran and focused on overweight or obese women of reproductive age, limiting the generalizability of the findings to more diverse populations.

The short duration of the interventions, generally between 8 and 12 weeks, might not be sufficient to observe their long-term effects or the sustainability of the benefits. Longer studies are needed to determine whether the improvements are maintained over time and to evaluate their impact on clinical outcomes such as menstrual regularity and fertility, also with probiotic doses ≥10^10^ CFU/day and without concurrent energy restriction [71].

The lack of comprehensive evaluations of the gut microbiota and body composition measurements limits the understanding of underlying mechanisms. Moreover, variability in doses, strains, and forms of the interventions makes it difficult to determine the most effective regimen and complicates comparisons between studies [29,72,73]. For example, some studies report an increase in *Bacteroides* spp. and *Lactobacillus* spp. in PCOS patients, while others highlight a decrease in *Prevotella* spp. and *Lachnospira* spp. [29,73]. This inconsistency in the bacterial strains studied and reported makes it challenging to identify specific microbial patterns associated with PCOS.

The absence of control and detailed reporting on dietary intake and physical activity is another limitation, as these factors can influence metabolic and hormonal parameters, introducing biases in interpretation.

### 4.5. Limitations of the Review

Despite efforts to conduct a thorough and rigorous systematic review, this research presents several limitations that should be considered when interpreting the results and planning future research in this field.

Firstly, there is the possibility of having excluded unpublished studies or studies published in languages other than English and Spanish. This review was based on searches in international databases and the literature accessible in these languages, which may have omitted relevant research published in other languages. This linguistic bias may limit the completeness of the evidence collected and lead to an overestimation or underestimation of the effects of the interventions.

Additionally, the inability to perform a quantitative meta-analysis due to heterogeneity among the included studies is a significant limitation. Differences in study designs, participant populations, interventions (types and doses of probiotics, prebiotics, and synbiotics), intervention duration, and outcome measures made it difficult to statistically combine the data. This heterogeneity impedes the quantitative synthesis of results and limits the ability to draw more general and robust conclusions about the efficacy of the interventions [29,73].

Another important limitation is the geographic and demographic homogeneity of the included studies. Most clinical trials were conducted in Iran and focused on overweight or obese women of reproductive age diagnosed with PCOS according to the Rotterdam criteria. This lack of diversity in the studied populations limits the generalizability of the findings to women of different ethnic, cultural, and socioeconomic backgrounds, as well as those with different clinical characteristics of PCOS.

Additionally, the variable methodological quality of the included studies represents a limitation. Although many studies presented low risk of bias in areas such as random sequence generation and allocation concealment, others showed risks in aspects like blinding, sample size, and follow-up duration. These methodological limitations can influence the internal validity of the studies and affect confidence in the reported findings.

Finally, the lack of standardized outcome measures and comprehensive evaluations of the gut microbiota hinders the comparison of studies and the understanding of underlying mechanisms. The bacterial strains and taxa examined vary across studies, with researchers often focusing on different bacterial families, genera, or species. Additionally, inconsistencies in methods of assessing insulin resistance, hormonal profiles, and inflammatory markers further complicate the ability to draw robust and comparable conclusions.

### 4.6. Clinical Implications

The findings of this systematic review suggest that synbiotic supplementation may be a valuable complementary strategy in the management of PCOS. Improvements in insulin resistance, lipid profiles, and hormonal balance indicate that synbiotics could address central metabolic and endocrine alterations of PCOS.

Variability in individual responses to probiotics and prebiotics highlights the importance of personalized treatment approaches. Factors such as initial gut microbiota composition, metabolic status, hormonal profiles, dietary habits, and genetic predispositions can influence the effectiveness of these interventions. Personalizing probiotic and prebiotic regimens according to each patient’s individual profile can maximize therapeutic outcomes [74,75,76].

Clinicians should consider the following factors [74,75,76,77,78]:

- Selection of specific strains: different probiotic strains can have varied effects on metabolic and hormonal parameters. Choosing strains with demonstrated efficacy could enhance treatment outcomes.
- Dosage and formulation: adjusting the dosage and choosing the appropriate formulation (capsules, powders, functional foods) according to patient preferences and tolerance can improve adherence and effectiveness.
- Comprehensive evaluation: assessing the patient’s overall health status, including metabolic markers, hormonal levels, and lifestyle factors, can help develop a tailored supplementation plan.

The personalization of synbiotic interventions could be especially beneficial for patients who have not responded adequately to standard treatments or who experience adverse effects from pharmacological therapies. Collaboration among healthcare professionals is essential to effectively implement personalized strategies [74,79,80].

Moreover, the management of PCOS often requires a multidisciplinary approach that addresses metabolic, reproductive, and psychological aspects. Synbiotic supplementation can be integrated into this care model as a complementary therapy. Healthcare professionals should educate patients about the potential benefits and limitations of probiotics and prebiotics, emphasizing that these supplements do not replace conventional treatments but can enhance overall management when used in conjunction [41,81].

Regular monitoring of metabolic and hormonal parameters is necessary to evaluate the effectiveness of the intervention and adjust treatment as needed. Promoting a balanced diet, regular physical activity, and adherence to medical therapies will optimize outcomes [41,82].

Although evidence supports the potential benefits of synbiotic supplementation, clinicians should be cautious due to variability in available products. They should consider the following factors:

- Quality and standardization: selecting high-quality products from reputable manufacturers is crucial, as efficacy depends on the viability and concentration of the strains used.
- Regulation: dietary supplements are not always subject to the same regulatory standards as medications. Clinicians should guide patients toward products proven in safety and efficacy.
- Patient education: informing patients about the importance of adherence, possible side effects, and realistic expectations will enhance satisfaction and compliance with the supplementation regimen.

Advances in microbiome research and personalized medicine could offer tools for more precise interventions. Microbiota profiling could identify specific dysbiosis patterns in women with PCOS, allowing for targeted probiotic and prebiotic therapies. Additionally, larger and longer clinical trials are required to establish standardized guidelines and determine optimal strains, doses, and durations of supplementation [41,83,84,85,86].

### 4.7. Recommendations for Future Research

More research is needed to address the current limitations. Future studies should be larger and multicentric randomized trials including diverse populations. Extending the duration of trials to six months or more will allow for the evaluation of the long-term efficacy and sustainability of the benefits.

Incorporating detailed analyses of the gut microbiota and metabolomic evaluations will help understand the biological mechanisms behind the observed clinical effects. Advanced techniques such as 16S rRNA gene sequencing and metagenomics can provide valuable information on how interventions alter microbial composition and metabolic pathways. Standardizing probiotic strains, doses, and administration forms will facilitate comparison between studies and the development of evidence-based clinical recommendations [87,88,89].

Including participants with different manifestations of PCOS will allow us to understand how interventions affect each subgroup and personalizing treatments. Evaluating clinical outcomes that directly impact quality of life, such as menstrual regularity and improvement of symptoms like hirsutism, is fundamental.

It is crucial to assess long-term safety and explore strategies to improve adherence, such as education and support programs. Investigating combination with other therapies may reveal additional benefits and optimize treatment protocols.

Conducting economic evaluations will help determine the value of these interventions compared to existing treatments, informing healthcare policy decisions. Incorporating patient perspectives in the study design will ensure that interventions address their needs, enhancing comprehensive care.

## 5. Conclusions

The studies reviewed in this review provide promising evidence on the use of synbiotics in the management of PCOS. The results indicate that synbiotic supplementation can significantly improve insulin resistance, lipid profiles, and hormonal balance in women with PCOS, suggesting a therapeutic potential to address the metabolic and endocrine alterations associated with this condition. These improvements can have a positive impact on the metabolic health and quality of life of patients, offering an alternative or complement to conventional therapies. However, greater validation is required through future research that addresses the current limitations.

Although the studies show encouraging results, the current body of evidence is limited by methodological constraints such as small sample sizes, homogeneity in the populations studied, relatively short intervention durations, and a lack of standardization in interventions and measurements. These limitations affect the generalization of the results and underscore the need to conduct additional studies with more robust and diversified designs. Only through larger, longer-term studies with standardized methodologies can the efficacy and safety of synbiotics in this population be confirmed, allowing for their evidence-based integration into clinical practice.

## Figures and Tables

**Table 1:** Eligibility criteria.

Criteria	Inclusion	Exclusion
**Study Design**	- Randomized controlled trials (RCTs) investigating supplementation with probiotics, prebiotics, or synbiotics in women with PCOS.	- Observational studies, reviews, cohort studies, case series, and studies with non-randomized designs.
		- Studies without an appropriate control group.
**Participants**	- Women diagnosed with PCOS according to the Rotterdam criteria, NIH criteria, or AES criteria.	- Studies including men or postmenopausal women.
	- Aged approximately 15–45 years (reproductive age).	
**Interventions**	- Supplementation with probiotics, prebiotics, or synbiotics administered in any form (e.g., capsules, fermented foods).	- Supplementation with compounds other than probiotics, prebiotics, or synbiotics (e.g., metformin alone without combination with probiotics).
**Duration**	- Interventions with a minimum duration of **8 weeks** to adequately assess changes in metabolic and hormonal parameters.	- Interventions with a duration of less than 8 weeks.
**Outcomes**	- Studies evaluating effects on insulin resistance (e.g., HOMA-IR, fasting insulin, fasting glucose), hormonal parameters (e.g., testosterone, SHBG, DHEA-S), and changes in other relevant metabolic markers (e.g., lipid profile, apelin, hs-CRP).	- Studies that only evaluated clinical outcomes such as reproductive function without assessing insulin resistance or hormonal parameters.
**Language**	- Studies published in English.	- Studies published in languages other than English.

**Table 2:** General characteristics of studies.

Study Reference	Country	Diagnostic Criteria	Study Design
Esmaeilinezhad et al., 2018 [38]	Iran	Rotterdam criteria for PCOS	Triple-blind RCT
Shoaei et al., 2021 [39]	Iran	Rotterdam criteria for PCOS	Double-blind RCT
Darvishi et al., 2020 [40]	Iran	Rotterdam criteria for PCOS	Double-blind RCT
Karimi et al., 2018 [45]	Iran	Rotterdam criteria for PCOS	Double-blind RCT
Samimi et al., 2018 [46]	Iran	Rotterdam criteria for PCOS	Double-blind RCT
Esmaeilinezhad et al., 2019 [37]	Iran	Rotterdam criteria for PCOS	Triple-blind RCT
Gholizadeh Shamasbi et al., 2018 [48]	Iran	Rotterdam criteria for PCOS	Triple-blind RCT
Arab et al., 2022 [49]	Iran	Rotterdam criteria for PCOS	Double-blind RCT
Karamali et al., 2018 [50]	Iran	Rotterdam criteria for PCOS	Double-blind RCT
Karimi et al., 2020 [51]	Iran	Rotterdam criteria for PCOS	Double-blind RCT
Nasri et al., 2018 [52]	Iran	Rotterdam criteria for PCOS	Double-blind RCT

**Table 3:** Participant characteristics.

Study Reference	Participants	Size	Age (Years)	BMI (kg/m^2^)	Baseline Parameters
Esmaeilinezhad et al., 2018 [38]	Women	I (pomegranate+synbiotic): 23, I (pomegranate): 23, I (synbiotic beverage): 23, C: 23	15–48	25–28	Synbiotic pomegranate juice: HOMA-IR: 6.32 ± 1.32 FBS: 112.04 ± 9.41 mg/dL Insulin: 22.80 ± 3.97 μIU/mL QUICKI: 0.294 ± 0.008; pomegranate juice: HOMA-IR: 6.16 ± 1.17 FBS: 112.82 ± 12.61 mg/dL Insulin: 22.15 ± 3.48 μIU/mL QUICKI: 0.295 ± 0.007; synbiotic beverage: HOMA-IR: 6.11 ± 1.22 FBS: 112.65 ± 8.46 mg/dL Insulin: 22.02 ± 4.32 μIU/mL QUICKI: 0.295 ± 0.008; control: HOMA-IR: 6.95 ± 0.91 FBS: 114.56 ± 8.16 mg/dL Insulin: 24.66 ± 3.33 μIU/mL QUICKI: 0.290 ± 0.004
Shoaei et al., 2021 [39]	Women	I (probiotic): 36 C (placebo): 36	15–40	25–30	Probiotic: HOMA-IR: 2.11 ± 0.21 FBS: 85.7 ± 2.6 mg/dL Insulin: 9.8 ± 0.9 μIU/mL QUICKI: N/A; placebo: HOMA-IR: 2.05 ± 0.23 FBS: 86.2 ± 2.5 mg/dL Insulin: 9.7 ± 0.8 μIU/mL QUICKI: N/A
Darvishi et al., 2020 [40]	Women	I (synbiotic): 34 C (placebo): 34	20–44	≥25	Synbiotic: HOMA-IR: 3.06 ± 1.35 FBS: 91.32 ± 8.07 mg/dL Insulin: 13.36 ± 4.89 μIU/mL HDL: 45.79 ± 12.05 mg/dL; placebo: HOMA-IR: 2.10 ± 1.12 FBS: 89.02 ± 9.05 mg/dL Insulin: 9.46 ± 4.64 μIU/mL HDL: 48.14 ± 10.22 mg/dL
Karimi et al., 2018 [45]	Women	I (synbiotic): 44 C (placebo): 44	19–37	≥25	Synbiotic: HOMA-IR: 3.77 ± 2.35 FBS: 92 ± 9 mg/dL Apelin 36: 27 ± 21 nmol/L CRP: 6.9 ± 5.99 mg/L; placebo: HOMA-IR: 3.6 ± 1.92 FBS: 90 ± 9 mg/dL Apelin 36: 26 ± 15 nmol/L CRP: 4.74 ± 4.68 mg/L
Samimi et al., 2018 [46]	Women	I (synbiotic): 30 C (placebo): 30	18–40	27–35	Synbiotic: FPG: 92.2 ± 6.2 mg/dL Insulin: 12.9 ± 4.2 μIU/mL HOMA-IR: 3.0 ± 1.1 Triglycerides: 146.4 ± 56.3 mg/dL VLDL: 29.3 ± 11.2 mg/dL AIP: 0.49 ± 0.20 Placebo: FPG: 94.0 ± 5.7 mg/dL Insulin: 12.1 ± 6.3 μIU/mL HOMA-IR: 2.8 ± 1.4 Triglycerides: 138.2 ± 37.9 mg/dL VLDL: 27.6 ± 7.6 mg/dL AIP: 0.44 ± 0.16
Esmaeilinezhad et al., 2019 [37]	Women	SPJ (synbiotic pomegranate juice): 23 PJ (pomegranate juice): 23 SB (synbiotic beverage): 23 PB (placebo beverage): 23	15–48	~25–28	SPJ: TGs: 171 ± 57 mg/dL TC: 180 ± 32 mg/dL LDL-C: 96 ± 35 mg/dL HDL-C: 50 ± 12 mg/dL SBP: 128 ± 7 mmHg Placebo: TGs: 194 ± 67 mg/dL TC: 194 ± 23 mg/dL LDL-C: 113 ± 27 mg/dL HDL-C: 42 ± 10 mg/dL SBP: 134 ± 7 mmHg
Gholizadeh Shamasbi et al., 2018 [48]	Women	I: 31 C: 31	18–45	25–40	Prebiotic: LDL-C: 106.87 ± 34.7 mg/dL HDL-C: 40.55 ± 8.8 mg/dL Total cholesterol: 166.90 ± 38.6 mg/dL TGs: 96.77 ± 35.7 mg/dL FBS: 80.68 ± 12.3 mg/dL hs-CRP: 4.70 ± 2.6 mg/dL Free testosterone: 1.25 ± 0.9 pg/mL DHEA-S: 3.18 ± 2.2 μg/mL
Arab et al., 2022 [49]	Women	I: 45 C: 43	15–40	≥25	Probiotic: SHBG: 36.11 ± 10.87 nmol/mL Total testosterone: 0.42 ± 0.14 ng/mL FAI: 3.24 ± 1.1 DHEA-S: 6.9 ± 2.8 nmol/L
Karamali et al., 2018 [50]	Women	I: 30 C: 30	18–40	≥25	Probiotic: SHBG: 46.3 ± 10.3 nmol/L Total testosterone: 1.3 ± 0.7 ng/mL mF-G scores: 14.1 ± 4.9 hs-CRP: 3546.7 ± 1003.1 ng/mL TAC: 935.5 ± 344.8 mmol/L MDA: 2.1 ± 0.4 μmol/L
Karimi et al., 2020 [51]	Women	I: 44 C: 44	19–37	≥25	Synbiotic: LDL: 97 ± 19 mg/dL HDL: 46.44 ± 7.69 mg/dL Total cholesterol (TC): 175.2 ± 27.5 mg/dL Triglycerides (TGs): 139 ± 78 mg/dL
Nasri et al., 2018 [52]	Women	I: 30 C: 30	18–40	≥25	Synbiotic: SHBG: 37.3 ± 13.1 nmol/L Total testosterone: 2.8 ± 1.3 ng/mL mF-G scores: 15.3 ± 5.6 hs-CRP: 2920 ± 2251.2 ng/mL NO: 39.0 ± 3.1 μmol/L MDA: 2.3 ± 0.4 μmol/L

Abbreviations: I = intervention group; C = control group; BMI = body mass index; HOMA-IR = Homeostatic Model Assessment of Insulin Resistance; FBS = fasting blood glucose; QUICKI = Quantitative Insulin Sensitivity Check Index; HDL-C = high-density lipoprotein cholesterol; LDL-C = low-density lipoprotein cholesterol; TGs = triglycerides; VLDL = very low-density lipoprotein; CRP = C-reactive protein; hs-CRP = high-sensitivity C-reactive protein; SHBG = sex hormone-binding globulin; FAI = free androgen index; DHEA-S = dehydroepiandrosterone sulfate; mF-G = modified Ferriman–Gallwey scores; TAC = total antioxidant capacity; MDA = malondialdehyde; NO = nitric oxide; SPJ = synbiotic pomegranate juice; PJ = pomegranate juice; SB = synbiotic beverage; PB = placebo beverage; FPG = fasting plasma glucose; N/A = not applicable; AIP = atherogenic index of plasma; SBP = systolic blood pressure.

**Table 4:** Intervention details and comparison groups.

Study Reference	Prebiotic, Probiotic, or Synbiotic Type	Pharmaceutical Form	Dosage	Duration	Comparison Group
Esmaeilinezhad et al., 2018 [38]	Synbiotic in pomegranate juice (SPJ)	Juice	2 L per week	12 weeks	Placebo pomegranate juice
Shoaei et al., 2021 [39]	Multistrain probiotic with *L. casei*, *L. acidophilus*, *L. rhamnosus*, *L. bulgaricus*, *B. breve*, *B. longum*, and *S. thermophilus*	Capsule	One 500 mg capsule daily	8 weeks	Placebo (starch and maltodextrin)
Darvishi et al., 2020 [40]	Synbiotic (*Lactobacillus casei*, *L. rhamnosus*, *L. bulgaricus*, *L. acidophilus*, *Bifidobacterium longum*, and *Streptococcus thermophilus*) and prebiotic (inulin and FOS)	Capsule	1 capsule daily, 500 mg	8 weeks	Placebo
Karimi et al., 2018 [45]	Synbiotic with 7 strains of probiotics (*L. acidophilus*, *L. casei*, *L. bulgaricus*, *L. rhamnosus*, *B. longum*, *B. breve*, and *S. thermophilus*) and prebiotic inulin (fructo-oligosaccharide)	Capsule	1 capsule daily, 1000 mg	12 weeks	Placebo
Samimi et al., 2018 [46]	Synbiotic with *L. acidophilus*, *L. casei*, *B. bifidum*, and 800 mg inulin	Capsule	2 × 10^9^ CFU/g of each strain + 800 mg inulin daily	12 weeks	Placebo
Esmaeilinezhad et al., 2019 [37]	Synbiotic in pomegranate juice (*Lactobacillus rhamnosus* GG, *Bacillus coagulans*, and *Bacillus indicus*)	Juice	300 mL daily	8 weeks	Placebo (flavored water)
Gholizadeh Shamasbi et al., 2018 [48]	Prebiotic (dextrin)	Powder (diluted in water)	20 g daily	12 weeks	Placebo (maltodextrin)
Arab et al., 2022 [49]	Multistrain probiotic with multiple strains: *Lactobacillus acidophilus* (3 × 10^10^ CFU/g), *Lactobacillus casei* (3 × 10^9^ CFU/g), *Lactobacillus rhamnosus* (1.5 × 10^9^ CFU/g), *Lactobacillus bulgaricus* (5 × 10^8^ CFU/g), *Bifidobacterium breve* (2 × 10^10^ CFU/g), *Bifidobacterium longum* (7 × 10^9^ CFU/g), *Streptococcus thermophilus* (3 × 10^8^ CFU/g) + 800 mg inulin	Capsule	One 500 mg capsule daily (7 strains + 800 mg inulin)	12 weeks	Placebo (starch and maltodextrin)
Karamali et al., 2018 [50]	Multistrain probiotic with multiple strains: *Lactobacillus acidophilus* (3 × 10^10^ CFU/g), *Lactobacillus casei* (3 × 10^9^ CFU/g), *Lactobacillus rhamnosus* (1.5 × 10^9^ CFU/g), *Lactobacillus bulgaricus* (5 × 10^8^ CFU/g), *Bifidobacterium breve* (2 × 10^10^ CFU/g), *Bifidobacterium longum* (7 × 10^9^ CFU/g), *Streptococcus thermophilus* (3 × 10^8^ CFU/g) + 800 mg inulin	Capsule	Two capsules daily (500 mg each: 7 strains + 800 mg inulin)	12 weeks	Placebo (starch and maltodextrin)
Karimi et al., 2020 [51]	Synbiotics with multiple strains: *Lactobacillus acidophilus* (3 × 10^10^ CFU/g), *Lactobacillus casei* (3 × 10^9^ CFU/g), *Lactobacillus bulgaricus* (5 × 10^8^ CFU/g), *Lactobacillus rhamnosus* (7 × 10^9^ CFU/g), *Bifidobacterium longum* (1 × 10^9^ CFU/g), *Bifidobacterium breve* (2 × 10^10^ CFU/g), *Streptococcus thermophilus* (3 × 10^8^ CFU/g) + inulin (fructooligosaccharide)	Capsules	Two capsules daily (500 mg each: 7 strains + inulin)	12 weeks	Placebo (starch and maltodextrin)
Nasri et al., 2018 [52]	Synbiotic with multiple strains: *Lactobacillus acidophilus* (2 × 10^9^ CFU/g), *Lactobacillus casei* (2 × 10^9^ CFU/g), *Bifidobacterium bifidum* (2 × 10^9^ CFU/g) + 0.8 g inulin	Capsules	Two 500 mg capsules daily (3 strains + inulin)	12 weeks	Placebo (starch and maltodextrin)

Abbreviations: SPJ = synbiotic pomegranate juice; CFU = colony-forming units; FOS = fructo-oligosaccharides.

**Table 5:** Results, adherence, and side effects.

Study Reference	Post-Intervention Parameters	Change in Parameters (Δ)	Comparative Effects	Adherence to the Intervention	Side Effects	Primary Outcomes	Secondary Outcomes	Measurement Methods	Key Findings	Author Conclusions	Study Limitations
Esmaeilinezhad et al., 2018 [38]	Synbiotic pomegranate juice: HOMA-IR: 5.75 ± 1.22; pomegranate juice: HOMA-IR: 6.20 ± 1.23; Synbiotic beverage: HOMA-IR: 5.61 ± 0.99 FBS: 111.47 ± 6.58 mg/dL Insulin: 20.36 ± 3.35 μIU/mL QUICKI: 0.29 ± 0.007 FBS: 113.68 ± 10.63 mg/dL Insulin: 22.07 ± 3.74 μIU/mL QUICKI: 0.29 ± 0.007 FBS: 110.36 ± 6.57 mg/dL Insulin: 21.03 ± 3.94 μIU/mL QUICKI: 0.29 ± 0.008; Control: HOMA-IR: 7.33 ± 0.92 FBS: 115.00 ± 7.85 mg/dL Insulin: 25.89 ± 3.11 μIU/mL QUICKI: 0.28 ± 0.004	Synbiotic pomegranate juice: ΔHOMA-IR: −0.57 ΔFBS: −1.68 mg/dL ΔInsulin: −1.77 μIU/mL; pomegranate juice: ΔHOMA-IR: +0.04 ΔFBS: +0.86 mg/dL ΔInsulin: −0.08 μIU/mL ΔQUICKI: 0.00; synbiotic beverage: ΔHOMA-IR: −0.50 ΔFBS: −1.18 mg/dL ΔInsulin: −1.66 μIU/mL ΔQUICKI: 0.00; Control: ΔHOMA-IR: +0.38 ΔFBS: +0.44 mg/dL ΔInsulin: +1.23 μIU/mL ΔQUICKI: −0.01	Synbiotic pomegranate juice: significant improvement (*p* < 0.05); pomegranate juice: no significant change; synbiotic beverage: moderate improvement (*p* < 0.05); control: no significant improvement	95% adherence, as most participants completed the study	None	Insulin resistance (HOMA-IR), fasting glucose	Testosterone, insulin sensitivity, lipid profile	ELISA for insulin and HOMA-IR, standard biochemical analysis	Significant reduction in HOMA-IR, increased insulin sensitivity, decreased testosterone	Synbiotic pomegranate juice improves insulin resistance and hormone levels in PCOS	Small sample size, lack of long-term follow-up
Shoaei et al., 2021 [39]	Probiotic: HOMA-IR: 1.9 ± 0.2 FBS: 81.5 ± 2.1 mg/dL Insulin: 9.3 ± 0.71 μIU/mL QUICKI: N/A Placebo: HOMA-IR: 2.00 ± 0.22 FBS: 88.3 ± 2.7 mg/dL Insulin: 9.8 ± 0.8 μIU/mL QUICKI: N/A	ΔHOMA-IR: probiotic: −0.21 vs. placebo: −0.05 ΔFBS: probiotic: −4.15 mg/dL vs. placebo: +2.57 mg/dL ΔInsulin: probiotic: −0.49 μIU/mL vs. placebo: +0.34 μIU/mL	Probiotic group showed non-significant changes in FBS, insulin, and HOMA-IR (*p* = 0.7); however, after adjusting for covariates, insulin reduction was significant in the probiotic group (*p* = 0.02)	90% adherence, most participants completed the study	None	Pancreatic β-cell function (FBS, serum insulin, HOMA-IR, QUICKI), CRP (C-reactive protein)	Insulin, lipid profile, hs-CRP	Standard biochemical analyses, immunoassay for insulin, HOMA-IR and QUICKI calculations	Non-significant reduction in FBS, serum insulin, and HOMA-IR in probiotic group; after adjusting for covariates, insulin reduction was significant; no significant differences in CRP	Probiotic supplementation for 8 weeks had a non-significant beneficial effect on pancreatic β-cell function and CRP	Short study duration, no glucose tolerance tests or hormonal evaluations
Darvishi et al., 2020 [40]	Synbiotic: HOMA-IR: 2.58 ± 1.15 FBS: 90.08 ± 7.90 mg/dL Insulin: 11.50 ± 4.75 μIU/mL HDL: 47.11 ± 12.73 mg/dL; placebo: HOMA-IR: 3.08 ± 1.31 FBS: 94.44 ± 9.49 mg/dL Insulin: 13.17 ± 5.29 μIU/mL HDL: 44.23 ± 10.73 mg/dL	ΔHOMA-IR: synbiotic: −0.47 vs. placebo: +0.98 ΔFBS: synbiotic: −1.24 mg/dL vs. placebo: +5.42 mg/dL ΔInsulin: synbiotic: −1.86 μIU/mL vs. placebo: +3.71 μIU/mL ΔHDL: synbiotic: +1.32 mg/dL vs. placebo: −3.91 mg/dL	Synbiotic group showed significant improvement in HOMA-IR, FBS, insulin, and HDL levels (*p* < 0.05) compared to placebo	95% adherence, all participants completed the study	None	Glycemic indices, lipid profile, obesity values	Serum apelin levels	Standard biochemical analysis, ELISA, anthropometric measurements	Significant improvements in glycemic indices, lipid profile, and obesity values; no changes in apelin	Synbiotic supplementation improves metabolic factors and obesity in women with PCOS	Short study duration, no evaluation of bacterial flora or SCFAs, only overweight/obese patients included
Karimi et al., 2018 [45]	Synbiotic: HOMA-IR: 3.82 ± 2.27 FBS: 92 ± 11 mg/dL Apelin 36: 14.4 ± 4.5 nmol/L CRP: 5.2 ± 3.9 mg/L; Placebo: HOMA-IR: 3.8 ± 2.46 FBS: 91 ± 10 mg/dL Apelin 36: 18.4 ± 9.2 nmol/L CRP: 4.9 ± 4.8 mg/L	ΔHOMA-IR: synbiotic: +0.05 vs. placebo: +0.2 ΔFBS: synbiotic: +0.6 mg/dL vs. placebo: +0.95 mg/dL ΔApelin 36: synbiotic: −12.6 nmol/L vs. placebo: −7.6 nmol/L ΔCRP: synbiotic: −1.7 mg/L vs. placebo: −0.24 mg/L	Synbiotic group showed a significant decrease in apelin 36 levels (*p* = 0.004) compared to placebo. No significant changes in metabolic parameters such as HOMA-IR, FBS, or CRP	Approx. 90% adherence, with 11 participants lost to follow-up	None	Metabolic parameters (fasting glucose, 2 h plasma glucose, HbA1c, HOMA-IR, QUICKI), fasting insulin, C-reactive protein (CRP), apelin 36 levels	QUICKI, CRP	Standard biochemical analysis, immunoturbidimetry for HbA1c and CRP, ELISA for apelin 36, HOMA-IR and QUICKI calculations	No significant differences in metabolic parameters, fasting insulin, or CRP after 12 weeks; significant decrease in apelin 36	Synbiotic supplementation had no significant effects on metabolic and inflammatory parameters; decrease in apelin 36	No examination of bacterial flora changes, potential reporting biases
Samimi et al., 2018 [46]	Synbiotic: FPG: 88.0 ± 7.2 mg/dL Insulin: 10.1 ± 3.9 μIU/mL HOMA-IR: 2.3 ± 0.9 Triglycerides: 130.3 ± 39.3 mg/dL VLDL: 26.0 ± 7.9 mg/dL AIP: 0.43 ± 0.16 Placebo: FPG: 92.8 ± 8.1 mg/dL Insulin: 13.9 ± 5.2 μIU/mL HOMA-IR: 3.2 ± 1.2 Triglycerides: 144.0 ± 47.2 mg/dL VLDL: 28.8 ± 9.4 mg/dL AIP: 0.43 ± 0.22	ΔFPG: synbiotic: −4.1 mg/dL vs. placebo: −1.2 mg/dL ΔInsulin: synbiotic: −2.8 μIU/mL vs. placebo: +1.8 μIU/mL ΔHOMA-IR: synbiotic: −0.7 vs. placebo: +0.4 ΔTriglycerides: synbiotic: −16.2 mg/dL vs. placebo: +5.8 mg/dL ΔVLDL: synbiotic: −3.3 mg/dL vs. placebo: +1.1 mg/dL ΔAIP: synbiotic: −0.05 vs. placebo: −0.003	Significant reduction in insulin, HOMA-IR, triglycerides, VLDL-cholesterol, and AIP in the synbiotic group (*p* < 0.05) compared to placebo. No significant differences observed in total cholesterol, LDL-cholesterol, or HDL-cholesterol	Approx. 95% adherence; 4 participants (2 from each group) were lost to follow-up due to personal reasons	None	Glycemic control markers (insulin, HOMA-IR, QUICKI)	Lipid profile (triglycerides, VLDL-C, AIP)	Standard biochemical analyses, ELISA for insulin, HOMA-IR and QUICKI calculations	Significant decrease in serum insulin, HOMA-IR, triglycerides, VLDL cholesterol, and AIP; significant increase in QUICKI in synbiotic group	Improvement in insulin resistance markers and some lipid parameters	Short follow-up, no SCFAs measured in stool
Esmaeilinezhad et al., 2019 [37]	SPJ: TGs: −26.4 mg/dL TC: −13.4 mg/dL LDL-C: −18.9 mg/dL HDL-C: +10.7 mg/dL SBP: −5.6 mmHg Placebo: TGs: +4.0 mg/dL TC: +4.3 mg/dL LDL-C: +7.2 mg/dL HDL-C: −3.7 mg/dL SBP: +1.5 mmHg	ΔTGs: SPJ: −26.4 mg/dL vs. placebo: +4.0 mg/dL ΔTC: SPJ: −13.4 mg/dL vs. placebo: +4.3 mg/dL ΔLDL-C: SPJ: −18.9 mg/dL vs. placebo: +7.2 mg/dL ΔHDL-C: SPJ: +10.7 mg/dL vs. placebo: −3.7 mg/dL ΔSBP: SPJ: −5.6 mmHg vs. placebo: +1.5 mmHg	Significant improvement in TGs, LDL-C, HDL-C, and SBP in the SPJ group compared to placebo. Increases in antioxidant capacity (TAC) and reductions in oxidative stress (MDA) were also noted	High adherence (≥90%); reminder messages were sent weekly, and empty bottles were returned to ensure compliance	None	Lipid profile, oxidative stress (MDA, TAC), hs-CRP, blood pressure	Not specified	Standard biochemical analysis, ELISA for hs-CRP, MDA and TAC measurements, blood pressure	Significant improvements in lipid profile, oxidative stress, inflammation, and blood pressure in SPJ, PJ, and SB groups compared to placebo	Synbiotic pomegranate juice improved metabolic, oxidative, and inflammatory outcomes	No measurement of gut microbiota changes or body composition
Gholizadeh Shamasbi et al., 2018 [48]	Prebiotic: LDL-C: 87.35 mg/dL HDL-C: 46.15 mg/dL Total cholesterol: 154.71 mg/dL TGs: 94.22 mg/dL FBS: 67.68 mg/dL hs-CRP: 3.11 mg/dL Free testosterone: 1.06 pg/mL DHEA-S: 2.77 μg/mL	ΔLDL-C: −29.79 mg/dL ΔHDL-C: +5.82 mg/dL ΔTotal cholesterol: −29.98 mg/dL ΔTGs: −38.50 mg/dL ΔFBS: −11.24 mg/dL Δhs-CRP: −1.75 mg/dL ΔFree testosterone: −0.32 pg/mL ΔDHEA-S: −0.7 μg/mL	Significant reduction in LDL-C, total cholesterol, triglycerides, FBS, hs-CRP, DHEA-S, and free testosterone in the prebiotic group compared to placebo. HDL-C increased significantly in the prebiotic group	High adherence (weekly follow-up calls ensured compliance)	Two participants experienced mild allergies and discontinued intervention	Lipid levels, fasting glucose, hs-CRP, DHEA-S, free testosterone	Hirsutism, menstrual irregularity	Standard biochemical analyses, ELISA for hormones, Ferriman–Gallwey scale	Significant decrease in LDL-C, total cholesterol, triglycerides, FBS, hs-CRP, DHEA-S, free testosterone, and hirsutism score; significant increase in HDL-C	Resistant dextrin regulates metabolic parameters and androgen levels in PCOS	Small sample size, participants only overweight/obese
Arab et al., 2022 [49]	Probiotic: SHBG: 40.06 ± 9.14 nmol/mL Total testosterone: 0.41 ± 0.15 ng/mL FAI: 3.22 ± 1.2 DHEA-S: 6.84 ± 2.9 nmol/L	ΔSHBG: +3.95 nmol/mL ΔTotal testosterone: −0.01 ng/mL ΔFAI: −0.02 ΔDHEA-S: −0.06 nmol/L	Probiotic supplementation significantly increased SHBG compared to the placebo group, but no significant changes were observed in total testosterone, FAI, DHEA-S, or clinical outcomes (acne, hirsutism)	High adherence: compliance monitored via phone calls, text messages, and capsule return	None	Hormonal and clinical parameters: SHBG, LH, FSH, DHEA-S, TT, FAI	Acne, hirsutism	Hormone profiles by electrochemiluminescence immunoassays, clinical signs evaluated by standardized scales	Significant increase in SHBG; no significant improvements in other hormonal or clinical parameters	Probiotic supplementation improved SHBG but not other hormonal or clinical parameters	Self-report instead of bacterial stool analysis, short duration
Karamali et al., 2018 [50]	Probiotic: SHBG: 72.2 ± 31.9 nmol/L Total testosterone: 1.1 ± 0.8 ng/mL mF-G scores: 12.4 ± 3.8 hs-CRP: 2396.7 ± 1588.6 ng/mL TAC: 948.3 ± 380.2 mmol/L MDA: 1.9 ± 0.6 μmol/L	ΔSHBG: +25.9 nmol/L ΔTotal testosterone: −0.2 ng/mL ΔmF-G scores: −1.7 Δhs-CRP: −1150 ng/mL ΔTAC: +8.8 mmol/L ΔMDA: −0.2 μmol/L	Probiotic supplementation significantly increased SHBG, decreased total testosterone, mF-G scores, hs-CRP, and MDA levels, and increased TAC compared to the placebo group. No significant effects on DHEA-S or other metabolic profiles	Compliance monitored via capsule count and daily SMS reminders	None	Hormonal and clinical parameters: SHBG, LH, FSH, DHEA-S, TT, FAI	Acne, hirsutism	Hormonal profile: electrochemiluminescence-based immunometric assays, biomarkers and clinical signs evaluated	Significant improvements in SHBG, decrease in total testosterone, and hs-CRP and TAC	Improvements in SHBG, testosterone, and inflammatory markers	Short duration, other strain combinations or prebiotics not evaluated
Karimi et al., 2020 [51]	Synbiotic: LDL: 92 ± 19 mg/dL HDL: 45 ± 8 mg/dL TC: 170 ± 24 mg/dL TGs: 141 ± 78 mg/dL	ΔLDL: −5.27 mg/dL ΔHDL: +1.71 mg/dL ΔTC: −5.2 mg/dL (not significant) ΔTGs: −2.2 mg/dL (not significant)	Synbiotic supplementation significantly decreased LDL levels and increased HDL levels compared to the placebo group. No significant effects were found for total cholesterol or triglycerides	Compliance monitored via capsule count and daily SMS reminders	None	Lipids and anthropometric measures: LDL, HDL, TC, TGs	Anthropometric indicators: weight, BMI, WC, HC, WHR	Lipid profile: TC, TGs, HDL measured by colorimetric methods, anthropometric indicators measured with digital scale	Significant decrease in LDL, increase in HDL; no differences in other anthropometric measures	Improvements in LDL and HDL, no changes in other parameters	Short duration limited to 12 weeks, dietary reporting biases
Nasri et al., 2018 [52]	Synbiotic: SHBG: 57.1 ± 48.6 nmol/L Total testosterone: 2.4 ± 0.9 ng/mL mF-G scores: 14.0 ± 4.9 hs-CRP: 1970 ± 1442.0 ng/mL NO: 44.5 ± 5.0 μmol/L MDA: 2.1 ± 0.4 μmol/L	ΔSHBG: +19.8 nmol/L ΔTotal testosterone: −0.4 ng/mL ΔmF-G scores: −1.3 Δhs-CRP: −950 ng/mL ΔNO: +5.5 μmol/L ΔMDA: −0.2 μmol/L	Synbiotic supplementation significantly increased SHBG, decreased mF-G scores, FAI, hs-CRP, and NO levels compared to the placebo group. No significant effects were found for other hormonal markers and biomarkers of oxidative stress	Compliance monitored via capsule count and daily SMS reminders.	None	Hormonal, inflammation, and oxidative stress: SHBG, LH, FSH, DHEA-S, TT, FAI	Inflammation biomarkers: hs-CRP	Hormonal profile: ELISA kits (DiaMetra, Italy), biomarkers: spectrophotometric methods for NO, TAC, GSH, MDA	Significant increase in SHBG, significant decrease in hs-CRP, NO, and mF-G scores	Synbiotics improved SHBG, NO, hs-CRP, and mF-G scores	Short duration, small sample size, no comparison of different combinations

Abbreviations: HOMA-IR = Homeostatic Model Assessment of Insulin Resistance; FBS = fasting blood glucose; QUICKI = Quantitative Insulin Sensitivity Check Index; HDL-C = high-density lipoprotein cholesterol; LDL-C = low-density lipoprotein cholesterol; TGs = triglycerides; VLDL = very low-density lipoprotein; CRP = C-reactive protein; hs-CRP = high-sensitivity C-reactive protein; SHBG = sex hormone-binding globulin; FAI = free androgen index; DHEA-S = dehydroepiandrosterone sulfate; mF-G = modified Ferriman–Gallwey scores; TAC = total antioxidant capacity; MDA = malondialdehyde; NO = nitric oxide; SPJ = synbiotic pomegranate juice; PJ = pomegranate juice; SB = synbiotic beverage; FPG = fasting plasma glucose; ELISA = Enzyme-Linked Immunosorbent Assay; SBP = systolic blood pressure; LH = luteinizing hormone; FSH = follicle-stimulating hormone; TT = total testosterone; WC = waist circumference; HC = hip circumference; WHR = waist-to-hip ratio; GSH = glutathione.

**Table 6:** Jadad scale assessment.

Study Name	Randomization (0–2)	Blinding (0–2)	Withdrawals/Dropouts (0–1)	Total Score (Out of 5)
Esmaeilinezhad et al., 2018 [38]	2	2	1	5
Shoaei et al., 2021 [39]	2	2	1	5
Darvishi et al., 2020 [40]	2	2	1	5
Karimi et al., 2018 [45]	2	2	1	5
Samimi et al., 2018 [46]	2	2	1	5
Esmaeilinezhad et al., 2019 [37]	2	2	1	5
Gholizadeh Shamasbi et al., 2018 [48]	2	2	1	5
Arab et al., 2022 [49]	2	2	1	5
Karamali et al., 2018 [50]	2	2	1	5
Karimi et al., 2020 [51]	2	2	1	5
Nasri et al., 2018 [52]	2	2	1	5

## Data Availability

Data are contained within the article.
